# Health disparities among Black deaf and hard of hearing Americans as compared to Black hearing Americans: A descriptive cross-sectional study

**DOI:** 10.1097/MD.0000000000028464

**Published:** 2022-01-14

**Authors:** Emmanuel Perrodin-Njoku, Carolyn Corbett, Rezenet Moges-Riedel, Laurene Simms, Poorna Kushalnagar

**Affiliations:** a Gallaudet University, Center for Deaf Health Equity, Washington, DC (currently at National Cancer Institute); b Gallaudet University, Department of Psychology, Washington, DC; c California State University at Long Beach, American Sign Language Linguistics and Deaf Cultures Program, Long Beach, CA.

**Keywords:** black, deaf, disparity, medical condition, sign language

## Abstract

There is a dearth of literature on health outcomes for Black people who identify as deaf or hard of hearing (DHH). Black DHH individuals generally experience at least 2 types of oppression, racism and audism, both of which contribute to health disparities within the Black and Deaf communities.

To understand the prevalence of health outcomes in a Black DHH adult sample and compare this to a Black hearing sample.

A descriptive cross-sectional study with primary Health Information National Trends Survey (HINTS)- American Sign Language survey data from Black DHH adults and secondary National Cancer Institute-HINTS English survey data from Black hearing adults.

Black DHH adults and Black hearing adults (18 years or older).

Using NCI's health information national trends survey in American Sign Language and English, self-reported data was gathered for all medical conditions as diagnosed by healthcare providers.

The study showed that Black DHH adults had a higher likelihood for diabetes, hypertension, lung disease, cancer, and comorbidity compared to their hearing Black counterparts.

Black DHH adults are at disparity for certain medical conditions compared to the general Black adult population. Future directions are needed to ensure that anti-racist policies include consideration of people with sensory disabilities. Inclusion of cultural and language needs of Black DHH patients in cultural humility training for healthcare providers is recommended to address health disparity in this population.

## 1 Introduction

Health disparities among the Black population in the United States are of an ongoing concern.^[1–4]^ However, very little is known about health disparities among Black individuals who self-identify as deaf or hard-of-hearing (DHH) and use American Sign Language (ASL) on a daily basis. There has been no formal way to actively identify individuals from this community. Census statistics typically combine Black DHH people within the Black population, or place them under the broad umbrella of individuals with disabilities, making it difficult to identify statistics unique to Black DHH signers.^[5]^ The prevalence and health estimates of Black DHH adults in the United States is virtually nonexistent, which results in overlooking the vital needs of this medically underserved group.

It is likely that the health disparities among Black Americans who are DHH and use ASL are greater than that of Black hearing Americans. Black DHH people generally experience at least 2 types of oppression: racism and audism.^[6]^ The term “racism” is defined as prejudice or discrimination against another with the belief that one's race is superior to the other person's. The term “audism” refers to the attitude and perspective that being able to hear is superior compared to being DHH.^[6,7]^ Due to their intersectional identities, Black DHH people may experience difficulties that are influenced by systemic racism and audism. Some examples may include, but are not limited to, difficulties with accessing equitable education, getting qualified interpreters in healthcare, and/or not having physicians who are not only language concordant in ASL but also self-identify as Black—in the US, there are no known Black DHH physicians that use ASL, as of this study's publication.

For Black people in the general population, mistrust was identified as one of many contributors to significant health disparities. According to a survey of active physicians, only 5% of the respondents self-identified as Black.^[8]^ The literature consistently notes that Black hearing individuals are more likely to encounter poorer communication quality with their doctors, be provided with less information, be given fewer opportunities to ask questions, and engage in less participatory medical decision-making compared to white patients.^[9]^ The issue of communication with one's doctor becomes more complicated when the patient is Black and DHH, and relies on ASL to access health information. It is not uncommon for Black DHH patients to bring a signing family member with them to medical appointments. These individuals are not interpreters and it is not appropriate practice for doctors to rely on them during medical appointments. When the patient is in need of a professional interpreter for a medical appointment, sign language interpreters may not always be available and may not meet the qualifications for medical interpreting certification. When available, the doctor may not be familiar with how to work with an interpreter. In addition, some medical practices rely on remote interpreting services, which reduces the opportunity for the interpreter and patient to establish a working rapport.^[10]^ It is also important to note that the power differential between doctor and patient remains, with the additional factor that the doctor is likely to be hearing. When working with an interpreter, the doctor may not take the additional time needed for ensuring that the patient understood the information that was being relayed, assuming that the interpreter has explained everything clearly and fully understood what the DHH patient signed.^[11]^

Implicit biases can also occur within medical settings and affect the quality of care for Black patients.^[12]^ When treating patients who are both Black and DHH, providers may experience implicit biases based on the patient's race, hearing status, and language difference. A recent study found that although medical professionals view themselves as not being biased, their implicit attitudes were biased against individuals with disabilities.^[13]^ For the purposes of this study, the medical needs of Black DHH adults will be viewed through the lens of their being a part of the Black population; that is, Black DHH adults are exposed to the same systemic racism that Black hearing adults are, which creates comparable rates of chronic health conditions.

As we consider the intersection of 2 medically underserved marginalized populations, the Black population and the DHH population, there is a scarcity of studies that include Black DHH people. Two previous studies pooled Black DHH individuals with other people of color rather than separating them into their own group.^[14,15]^ Ten studies reported the number of Black Deaf participants, however, they did not analyze data from the Black DHH subgroup alone due to small sample sizes.^[16–23]^ We do not know how many Black DHH Americans experienced a lifetime diagnosis of chronic medical conditions such as hypertension, diabetes, or cancer. This lack of information highlights the need for research to understand the state of health for Black DHH Americans and how they compare to Black hearing Americans. This paper seeks to:

1. describe the lifetime prevalence for certain medical conditions within a sample of Black DHH Americans, and
2. compare their lifetime prevalence rates with Black hearing Americans.

## 2 Methods

### 2.1 Study design and survey questionnaire

The institutional review board at Gallaudet University approved this study. In a descriptive cross-sectional study of Black adults in the United States, self-reported data was gathered from Black hearing adults who answered a set of questions about their medical conditions (hypertension, diabetes, lung disease/asthma/emphysema, bronchitis, cirrhosis/kidney problems, stroke, depression/anxiety, heart condition, arthritis/rheumatism, cancer) from the National Cancer Institute's Health Information National Trends Survey [https://hints.cancer.gov/; Health Information National Trends Survey]. They also answered questions related to demographics, health indicators, and patient centered communication in the same survey. Black DHH adult signers answered the same set of Health Information National Trends Survey questions that was translated to ASL and presented through a bilingual ASL/English online survey platform (see Kushalnagar et al., for more information).^[24]^ Online survey instead of face to face interviews was chosen to reduce potential sources of bias.

### 2.2 Recruitment procedure

After the Gallaudet University Institutional Review Board approved the study procedures, the research staff began recruitment through national channels, targeting the DHH community members who use ASL. Purposive sampling was used to ensure adequate representation with respect to key demographic characteristics such as race, age and education. Several approaches were used for recruiting DHH signers across the USA, including Hawaii and Alaska: snowball sampling through personal networks, distributing flyers, and advertising on deaf-centered organization websites and e-newsletters. Communication between the research staff and participants occurred through mail, email, social media, and video chat programs. Research staff provided prospective participants with an information flyer, and discussed the study purpose and procedures, reviewed inclusion and exclusion criteria, and answered any questions they might have had to determine eligibility and interest. Participants were told that they would receive a $25 valued gift card as a thank you for their participation. Individuals who self-reported that they used ASL as their primary language were included in the study. Individuals who were 17 years old or younger, as well as those who had unilateral hearing loss were excluded. Participants provided signed informed consent before entering the study. The survey took approximately 1 hour to complete. No names or identifying information were collected as part of this online survey. Each participant received a gratuity for participating in the study.

### 2.3 Statistical analyses

Data analyses were conducted using SPSS version 27. Descriptive statistical analysis, including percentages, was used to describe the prevalence of medical conditions in the Black DHH community that uses ASL. Chi-Squared analyses were conducted to test for equality of the proportions of various covariates between the DHH and hearing samples. Via multiple logistic regression analyses using a *P* value less than .05 for significance, hearing status (deaf vs hearing) was entered as predictor with medical conditions as outcomes. In these analyses, demographic (age, gender, education) and health (patient-centered communication, health status, BMI, smoking, and regular provider) variables were entered as covariates. Nonresponses were treated as missing data.

## 3 Results

### 3.1 Characteristics of the deaf and hearing samples

A total of 204 Black DHH ASL users and 531 Black hearing English speakers answered questions about medical conditions and demographics relevant to this study. Table 1 lists the frequency percentages for the age-weighted samples. A majority of both deaf and hearing samples had health insurance (96% and 94%, respectively) and a regular provider (65% and 63% respectively). Despite the similarity in health insurance rates between the 2 groups, Black DHH rated their physicians as having lower patient-centered communication skills than Black hearing participants (DHH, Patient centered communication Mean = 60.99 [SD = 25.89]; Hearing, Patient centered communication Mean = 82.25 [SD = 21.14]; *t test* = 16.97, *P* value < .001). Chi-Squared analyses showed a significant group difference only for lung disease and heart conditions: self-reported prevalence of lung disease was higher in the Black DHH group (19%) than in the Black hearing group (13%; X^2^ = 4.49, *P* < .05). The opposite was observed for heart conditions with the self-reported prevalence being significantly higher in the Black hearing group than the Black DHH group (X^2^ = 3.81, *P* = .05). For all other medical conditions, both groups had similar prevalence rates.

**Table 1:** Sociodemographic characteristics for Black deaf and hearing adult samples.

		Black deaf ASL speakers		Black hearing english speakers	
Variable	N = 204	%	N = 531	%	*X*^2^ (*P* value)
Hypertension					9.17^∗∗^ (.002)
Yes	87	44.2	297	55.9	
No	110	55.8	226	42.6	
Diabetes					1.95 (.162)
Yes	41	20.9	135	25.4	
No	155	79.1	385	72.5	
Lung Disease					4.40^∗^ (.036)
Yes	39	19.9	71	13.4	
No	157	80.1	452	85.1	
Cancer					1.84 (.175)
Yes	12	6.3	50	9.4	
No	180	93.8	479	90.2	
Depression or anxiety disorder					0.08 (.775)
Yes	38	19.4	106	20.0	
No	158	80.6	415	78.2	
Heart condition					0.62 (.252)
Yes	17	8.3	52	9.8	
No	178	87.3	470	88.5	
Arthritis					9.05^∗^ (.003)
Yes	39	20.6	168	31.6	
No	150	79.4	353	66.5	
BMI					1.98 (.577)
Underweight	2	1.4	10	1.9	
Normal	37	26.2	103	20.0	
Overweight	51	36.2	162	31.5	
Obese	51	36.2	239	46.5	
Education					1.91 (.386)
No college degree	120	58.8	338	63.8	
Have a college degree	84	41.2	192	36.2	
Age					87.19 (.001)
18-34	85	41.7	61	11.5	
35-49	51	25.0	142	26.7	
50-64	50	24.5	214	40.3	
65-74	13	6.4	70	13.2	
75+	5	2.5	33	6.2	
Gender					1.24 (.266)
Male	75	37.1	166	32.7	
Female	127	62.9	341	67.3	

∗ *P* < .05.

∗∗ *P* < .01. ^∗∗∗^*P* < .001. Note: some numbers are uneven due to missing data.

As shown in Table 2, multiple logistic regression analyses were performed to assess the relationship between hearing status and health outcomes after controlling for demographic and health correlates. Compared to Black hearing counterparts, Black DHH adults had a higher likelihood for the following health conditions: diabetes (OR = 1.77, 95% CI = 1.04, 3.02), hypertension (OR = 1.73, 95% CI = 1.11, 2.72), lung disease (OR = 1.72, 95% CI = 1.00, 2.98), cancer (OR = 3.52, 95% CI = 1.61, 1.71), and comorbidity (OR = 2.91, 95% CI = 1.77, 4.81). There were no group differences for heart disease, arthritis, depression/anxiety, and obesity.

**Table 2:** Summary of multiple binary logistic regression analyses predicting the association between hearing status and health outcomes^a^.

	Diabetes	Hypertension	Heart disease	Lung disease	Arthritis	Depression/anxiety	Cancer	Comorbidity	Obesity
	aOR (95% CI)	aOR (95% CI)	aOR (95% CI)	aOR (95% CI)	aOR (95% CI)	aOR (95% CI)	aOR (95% CI)	aOR (95% CI)	aOR (95% CI)
Hearing status^c^	**1.77** ^∗^ **(1.04, 3.02)**	**1.73** ^∗^ **(1.11, 2.72)**	2.01 (0.94, 4.32)	**1.72**^∗^ (1.00, 2.98)	1.50 (0.86, 2.60)	1.05 (0.64, 1.74)	**3.52** ^∗∗^ **(1.61, 7.71)**	**2.91** ^∗∗∗^ **(1.77, 4.81)**	0.83 (.56,1.23)
Age^b^	**1.05** ^∗∗∗^ **(1.04, 1.07)**	**1.07** ^∗∗∗^ **(1.06, 1.09)**	**1.04** ^∗∗∗^ **(1.02, 1.06)**	.997 (0.98, 1.01)	**1.07** ^∗∗∗^ **(1.05, 1.09)**	1.00 (0.99, 1.02)	**1.06** ^∗∗∗^ **(1.03, 1.08)**	**1.08** ^∗∗∗^ **(1.07, 1.10)**	1.00 (0.99, 1.01)
Male^c^	0.93 (0.63, 1.37)	0.60 (0.42, 0.84)	0.79 (0.54, 1.59)	**2.21** ^∗∗∗^ **(1.40, 3.48)**	**1.89** ^∗∗^ **(1.25, 2.86)**	1.33 (0.90, 1.96)	**1.96**^∗^**(1.03, 3.71**)	1.32 (0.92, 1.89)	**1.92** ^∗∗∗^ **(1.42, 2.61)**
Education^c^	1.00 (0.68, 1.48)	1.54 (0.42, 0.84)	1.78 (0.98, 3.22)	1.10 (0.73, 1.67)	**1.73** ^∗∗^ **(1.16,2.60)**	1.24 (0.85, 1.82)	1.19 (0.67, 2.12)	**1.57** ^∗^ **(1.10,2.25)**	1.10 (0.81, 1.49)
Health status^c^									
Poor/Fair	**4.12**^∗∗^ (**2.48, 6.79**)	**1.89** ^∗∗^ **(1.21, 2.96)**	**3.15** ^∗∗∗^ **(1.69, 5.88)**	**3.73** ^∗∗∗^ **(2.22, 6.29)**	**2.48** ^∗∗∗^ **(1.49, 4.12)**	**3.54** ^∗∗∗^ **(2.19, 5.70)**	**2.36** ^∗^ **(1.05, 5.30)**	**5.46** ^∗∗∗^ **(3.38, 8.82)**	**2.59** ^∗∗∗^ **(1.72, 3.92)**
Good	**2.10**^∗∗∗^ (**1.34 3.30**)	**1.68** ^∗∗^ **(1.17, 2.41)**	0.67 (0.34, 1.34)	1.29 (0.80, 2.09)	1.55 (0.99, 2.41)	1.05 (0.64, 1.74)	**2.66** ^∗∗^ **(1.32, 5.33)**	**1.94** ^∗∗∗^ **(1.32, 2.85)**	**1.59** ^∗∗^ **(1.15, 2.21)**
Regular provider^c^	0.79 (0.51, 1.22)	**0.60** ^∗∗^ **(0.43, 0.86)**	**0.46** ^∗^ **(0.23, 0.91)**	**0.56** ^∗^ **(0.35, 0.89)**	**0.50** ^∗∗^ **(0.32, 0.79)**	**0.53** ^∗∗^ **(0.35, 0.82)**	0.55 (0.27, 1.11)	**0.57** ^∗^ **(0.39, 0.84)**	.74 (0.54,1.02)
Patient-centered communication^b^	1.00 (.997, 1.01)	1.00 (0.999, 1.01)	1.00 (0.99, 1.01)	1.00 (.997,1.01)	1.00 (0.99, 1.01)	1.00 (0.99, 1.01)	0.997 (0.99, 1.01)	**1.01** ^∗^ **(1.00, 1.01)**	**1.01** ^∗∗^ **(1.00, 1.01)**

a The comparison group is no medical outcome, no comorbidity, or not obese.

b Age and patient centered communication are continuous variables.

c Reference groups: Hearing, female, college degree, and have a regular provider.

∗∗∗ = *P* < .001.

∗∗ = *P* < .01.

∗ = *P* < .05.

For both the DHH and hearing samples, Table 2 shows a strong association between having a regular provider and most self-reported medical conditions. Older age is also associated with having a medical condition. People who perceive their health status to be poor are much more likely to report having a medical condition compared to people who perceive their health status as excellent.

## 4 Discussion

To our knowledge, this is the first study that reports the lifetime prevalence of medical conditions among Black DHH Americans and compares the group with Black hearing Americans. Historically, members of the Black community have approached the prospect of medical intervention with significant trepidation. The issue of medical mistrust is also a concern for members of the Black DHH community, as well as for DHH people who do not self-identify as Black.^[15,25]^ Jaiswal & Halkitis highlighted the issue of medical mistrust, and asserted that it is a major factor in the lower utilization of health care services as well as overall management of long-term health conditions in minority communities.^[26]^ For both DHH and hearing Black participants, our study shows a connection between having a medical condition and not having a regular provider, an indirect measure of low utilization of health care services. Education may serve to reduce the impact of mistrust on poor health outcomes,^[27]^ with college-educated individuals more likely to seek out preventive care and physical exams than people who do not have a college degree.^[28]^

For Black people who are DHH, education alone is not enough to ensure full access to and utilization of health care services. Individuals from this population need to also have access to incidental information related to health, and this information is often inaccessible through spoken language-dominated media and television. In a recent study with approximately 800 DHH respondents, DHH adults’ ratings of their providers’ patient-centered communication behaviors were associated with the DHH respondents’ perception of the interpreters’ receptive sign language skills.^[11]^ The findings of this study raise an important implication for people who self-identify as both Black and DHH. When the interpreter is white and does not have strong receptive skills nor understanding of Black ASL,^[29]^ there is the potential for disruption and a possible negative impact on the Black DHH patient's relationship with their physician. If this is the case, then this would explain the significantly lower Patient-Centered Communication score among Black DHH respondents compared to Black hearing respondents.

The current study found that there were significant differences in the prevalence of many medical conditions diagnosed by healthcare providers when Black DHH participants and Black hearing participants were compared, even after controlling for age and education. There were poorer health outcomes for Black DHH in the following medical conditions: diabetes, hypertension, heart condition, lung disease, and cancer, as well as comorbidity.

Our study found that, compared to Black hearing participants, Deaf Black participants were almost 2 times more likely to report having diabetes and 3 times more likely to report themselves as having comorbidities. Studies of the general population show that type 2 diabetes mellitus (T2DM) is the most common type of diabetes in US adults, with an estimated 90% of all cases of diabetes being a result of T2DM.^[30]^ The high prevalence of diabetes in the US population disproportionately affects minority populations, with non-Hispanic Blacks having higher rates of incidence and mortality due to T2DM-related conditions.^[31,32]^ With literature showing a clear, consistent disparity in the prevalence of diabetes between hearing Black people as well as those in other ethnic and racial minority groups, compared to hearing white people, it stands to reason that Black DHH people are at increased risk of being diagnosed with diabetes and other comorbidities stemming from diabetes due to race and being a linguistic minority. Using ASL is associated with extrinsic factors such as accessibility and attitudinal barriers to quality healthcare, hence the greater prevalence of medical conditions among Black DHH adults compared to Black hearing adults.

The greater disparity in hypertension among Black DHH people compared to hearing Black people is not surprising, given the finding in a previous study that reported higher likelihood of hypertension among Black DHH people.^[33]^ While the finding was consistent with general literature documenting higher prevalence of hypertension among Black Americans compared to other races, our study shows a clear disparity in the Black DHH adult population compared to the Black hearing adult population. We believe that the DHH-specific disparity in hypertension is largely driven by multiple systemic factors, including oppressive attitudes toward DHH people due to race and hearing status, as well as barriers to optimal health care communication between DHH patients and their providers. In addition, obstacles to obtaining accessible information regarding preventative factors, dietary requirements, and food accessibility also contribute to this disparity.^[34]^

Literature shows that Black young adults already have a higher likelihood of developing a heart disease.^[35]^ If the Black adult is also DHH and experiences adverse childhood communication while growing up (e.g., DHH person and caregiver are unable to understand each other at all), the chances of developing a heart disease magnifies.^[36]^ The disparity in heart disease, especially among Black DHH, is of serious concern and can be prevented through both early intervention services and health advocates from within the Black DHH community.

Lung disease is much more prevalent among Black DHH participants compared to their hearing counterparts in this study. Additional research is needed to clarify factors that may drive the disparity between Black DHH and Black hearing participants. The authors of this study speculate that the higher self-reported prevalence of lung disease among Black DHH people may be associated with the ways that those individuals cope with stress, such as smoking behaviors. The literature confirms that cigarette smoking advertising is often specifically designed for communities of color, and more likely to be visually prevalent in the environment in minority communities.^[37]^ As stated previously, information about the dangers of smoking is often presented in written or spoken English, which limits access by DHH individuals.

The strong likelihood of self-reporting a cancer diagnosis among Black DHH people compared to Black hearing people in our study is a public health concern. In a study of DHH and hearing women's adherence to preventive screenings for cervical and breast cancer, Black DHH women generally have higher mammogram and pap smear adherence compared to non-Black DHH women.^[38]^ However, this study also showed that DHH women had much lower adherence to pap smear examinations compared to hearing women. If a person is DHH, Black, and female, the risk for cancer is magnified among those who do not have a regular provider, as well as those who have limited education. Serious concerns regarding cancer screening can also been seen in DHH men. In a shared decision-making and prostate-specific antigen (PSA) screening study, DHH male participants were more at risk of reporting that their doctors often did not engage them in making decisions about their health compared to hearing male participants.^[39]^ This study also reported a significantly higher percentage of white DHH men who received prostate-specific antigen screenings compared to Black DHH men, which must be interpreted with caution given the small number of Black DHH male respondents. Even though the number of cancer studies that included Black DHH people in the analysis is limited, the findings are clear that Black DHH people are not given enough attention or encouraged to receive age-appropriate cancer screenings. This is an area of opportunity for public health effort in both community and health care settings in order to increase cancer screening participation rates and more accurately understand the prevalence of cancer diagnoses, and to increase shared decision making.

The lack of association between depression/anxiety and hearing status in our current Black sample is surprising and adds to the inconsistency of the recent report of lower prevalence of self-reported depression/anxiety disorder among Black DHH adults (7.8%) compared to Black hearing adults (12.5%).^48^ It is possible that our current sample consisted of Black DHH participants who were not as isolated, which has been reported to be strongly associated with increased feelings of depression and anxiety.^[40]^ The question of whether mental health outcome disparities exist between Black DHH and Black hearing adults warrants further exploration, considering the effects of systemic and individual discrimination as well as stigma discouraging mental health professional help.

### 4.1 Limitation and future directions

Although this study utilized snowball sampling that has been found to be quite effective in recruiting and retaining members of hard-to-reach communities^[41]^ such as in our Black DHH sample, potential self-selection bias may be present. Our statistical findings are strong, and it is possible that the results will not change drastically with a larger sample size. Nevertheless, the expansion of the Black DHH sample would provide greater insights on whether the health disparity may be largely driven by adverse childhood communication experiences, which have been found to strongly predict certain medical conditions later in life.^[36]^ Another area that warrants attention in future research is the intersectionality of multiple identities among Black DHH people. The intersection of marginalized identities, including sexual orientation and gender identity, has been found to contribute to poorer health outcomes among DHH people who hold multiple marginalized identities.^[42,43]^ Further research into the prevalence of health outcomes between Black DHH and hearing individuals who hold multiple marginalized identities may inform the impact of intersectionality on the health outcomes of DHH people, of which there is a very limited body of literature.

### 4.2 Recommendations for effective delivery of healthcare to the black DHH community

Given the clear disparity in cancer diagnosis among Black DHH adults compared to their hearing counterparts, additional efforts will be necessary to eliminate cancer health disparities. Working through community leaders such as the National Black Deaf Advocates (www.nbda.org) could help explore and implement community-driven solutions in place to improve knowledge about cancer health, screening adherence, and overall care. Integrating social care such as community health workers into the delivery of cancer health care will be critical in promoting cancer health literacy, regular visits with providers, and screening adherence.

Tailored curriculum and training are needed to support sign language interpreters who interact with Black DHH patients as well as healthcare providers who encounter Black DHH patients in their care. Training should at minimum include content related to:

1. language and customs specific to the Black DHH community, and
2. understanding adverse childhood communication experiences and their relationship with adulthood chronic health conditions in the adult DHH population.^[36,44]^

DHH patient education for all health professionals must become a national mandate. Such a mandate will assist with stimulating medical education and training for healthcare professionals to become well informed about the diverse cultural and language needs of not only Black DHH patients but also those from other marginalized groups. Developing equitable and anti-racist policies that include consideration of people with sensory disabilities will also help push towards health equity.

The Black Hearing, Black DHH, and agencies that serve the DHH community (interpreting, religious, and social service) as a collective group can represent a large degree of cultural community wealth. This coordination remains untapped, but is a large resource, when combined, that can have a tremendous impact on the health trajectory of the Black DHH community.

## Acknowledgments

We wish to thank Dr. Nicole Singh as a reader. We thank Franklin Jones, Brenda Perrodin and family, and the National Black Deaf Association for their assistance with spreading the word about this study.

## Author contributions

**Conceptualization:** Emmanuel Perrodin-Njoku, Carolyn Corbett, Rezenet Moges-Riedel, Laurene Simms, Poorna Kushalnagar.

**Data curation:** Poorna Kushalnagar.

**Formal analysis:** Poorna Kushalnagar.

**Funding acquisition:** Poorna Kushalnagar.

**Investigation:** Emmanuel Perrodin-Njoku, Poorna Kushalnagar.

**Methodology:** Poorna Kushalnagar.

**Project administration:** Poorna Kushalnagar.

**Supervision:** Poorna Kushalnagar.

**Visualization/data presentation:** Poorna Kushalnagar.

**Writing – original draft:** Emmanuel Perrodin-Njoku, Poorna Kushalnagar.

**Writing – review & editing:** Carolyn Corbett, Rezenet Moges-Riedel.
